# Influence of Temperature and Saline Conditions on Bacteria Naturally Associated With the Cnidarian Host *Nematostella vectensis*

**DOI:** 10.1155/ijm/4107949

**Published:** 2025-11-21

**Authors:** Quinton Krueger, Madisun Shore, Adam M. Reitzel

**Affiliations:** ^1^Department of Biological Sciences, University of North Carolina at Charlotte, Charlotte, North Carolina, USA; ^2^Computational Intelligence to Predict Health and Environmental Risks, University of North Carolina at Charlotte, Charlotte, North Carolina, USA

**Keywords:** cnidarian, heat stress, microbiome, salinity, thermal tolerance

## Abstract

The associated microorganisms (“microbiome”) of multicellular individuals (“host”) are important for the physiology and survival of the host. Individual bacterial species vary in environmental tolerances that may limit their associations with hosts, especially when their range of survivable conditions is narrower. To elucidate the roles for different environmental niche spaces of bacteria that may compose the microbiome, we evaluated the survival and growth of individual and combinations of bacteria with and without an animal host, the sea anemone *Nematostella vectensis* (Cnidaria, Anthozoa). We assessed 62 environmental bacteria from seven genera (*Alteromonas*, *Bacillus*, *Grimontia*, *Photobacterium*, *Pseudoalteromonas*, *Shewanella*, and *Vibrio*) isolated from six estuaries and the host to determine their tolerance across a gradient of temperatures (30°–40°C) and salinities (5–30 ppt). Growth rates and plate counts revealed members of the *Vibrio* genus had the highest growth rate at higher salinities (15 and 30 ppt), while *Bacillus* and *Alteromonas* spp. exhibited consistent growth over a broader range of salinities and temperatures. Only 15% of isolates were capable of growth at the combination of highest temperature and lowest salinity (40°C, 5 ppt), suggesting that these environmentally relevant conditions may limit microbiome diversity. We further assessed three isolates (*Bacillus velezensis*, *Pseudoalteromonas spiralis*, and *Vibrio diabolicus*) for how bacterial growth changed when associated with *N. vectensis*. When anemones were exposed to environmentally relevant heat stress over 3 days, bacterial concentrations varied significantly. *P. spiralis* grew more under lower salinities and maintained stable concentrations. Conversely, *V. diabolicus* grew more with higher salinity and maintained these high concentrations in nearly all conditions. At sustained extreme temperatures for the anemones, the microbial composition exerted a small impact on survival. Together, these results support that environmental conditions are important drivers for the relative abundance of particular bacteria in the context of the host's microbiome.

## 1. Introduction

The composition of microbes associated with a host (microbiome) is dependent on a variety of abiotic (e.g., temperature [1, 2] and salinity [3, 4]) and biotic factors (e.g., the host organism [5] and bacteria–bacteria interactions [6–11]). These factors independently and in concert can influence the diversity and abundance of microbes present with a host species over time. The potential for bacteria to survive and replicate in an environment is a critical factor that determines if a particular species may constitute part of a host's microbiome, regardless of host immune responses. Bacteria can have variable tolerances, which impacts their survival in highly dynamic and extreme environments, where abiotic-tolerant species may be equipped to maintain colonization in animals that occupy similar environments. Thus, the occurrence of bacterial species in a host's microbiome may be due to their physiological tolerances (e.g., temperature, oxygen, and salinity) in addition to interaction with the host species. This expectation is consistent with the Baas Becking hypothesis where “everything is everywhere but the environment selects” [12]. Tolerant bacteria may be beneficial to a host species, where the association with the host may increase the holobiont's survival while under stress [13–15]. The synergisms of the host and microbiome may expand the environments in which both organisms can survive [10, 16]. Sensitivity to abiotic factors may explain the neutrality of the microbiome as part of the holobiont, as the community changes after passing through environmental filters [17]. Therefore, it is important to identify the physiological tolerances for individual bacteria that compose host-associated microbial communities to determine which are capable of surviving or thriving in different environments [18].

Numerous studies have shown that bacteria and microbial communities can be critical for the health and survival of marine invertebrate species [19, 20]. For example, the composition and function of the microbiome shift under heat stress in marine invertebrates [21–23], while increased temperatures can also induce pathogenicity of certain bacterial species [24]. Bacteria that can extend the physiological tolerances of the host, while also providing antagonistic properties against other bacteria, may be suitable candidates for probiotic applications in marine invertebrates [25–27]. While these characteristics have been determined for a small number of bacteria, the bacteria that are associated with marine invertebrates and their specific physiological tolerances are generally not studied in this context. Individual bacteria, such as species in the *Pseudoalteromonas* and *Bacillus* genera, promote the growth and presence of other probiotics [28]. In addition, bacteria can modulate immune responses in marine invertebrates and protect against future pathogen exposure, including *Vibrio* species [29–32]. Thus, individual bacteria can play a vital role in mechanisms through the life stages of the host, which necessitates further investigation into these interactions. To better understand the impacts on host survival, assessment of the diversity of physiologies of associated bacteria is required to understand these interactions.

*Nematostella vectensis* has developed into an experimental system for characterization of variation and potential function of the microbiome in cnidarians. Like many cnidarians, the microbiome for this anemone is composed of a variety of species that have been primarily identified through sequence-based approaches [7]. The microbiome of *N. vectensis* can vary due to seasonal shifts [33], daily variability [34], and by geographic location [35]. *N. vectensis*, like other marine organisms, has several life stages, and the microbiome changes across these distinct developmental phases [35]. Colonization of the microbiome is dependent on both the host–bacteria interactions and the bacteria–bacteria interactions [36]. The microbiome also confers increased physiological tolerance to the anemone host. In long-term laboratory experiments, the microbiome selected through high temperature can confer thermal tolerance to naive juvenile *N. vectensis* and their acclimated offspring [6]. Which members of these stress-selected communities specifically confer this resilience have not yet been characterized to determine how individual species or specific molecular products drive this acclimation. This bacteria-mediated acclimation may be important for survival of this species in their native environments. The estuaries that *N. vectensis* inhabit experience extreme water temperature fluctuations daily, up to 20°C [37]. *N. vectensis* can survive at 40°C for several hours, but prolonged exposure will lead to death [37].

There are little data on how individual bacteria species may be related to the biology of this anemone host. Different bacteria species (e.g., spirochetes) associate with different regions of the adult body, suggesting potential for different functions [38]. Colonization of *N. vectensis* by particular bacterial species is related to a variety of metabolic processes, including chitin synthesis as well as amino acid, nucleotide, and carbon metabolism [36]. To date, only one marine bacterium has been shown to be pathogenic against *N. vectensis*, *Vibrio coralliilyticus*, at temperature conditions above 30°C [39], although this species is not reported to naturally occur with *N. vectensis*. Because microbes can be important facilitators for the health and survival of *N. vectensis* like other species, assessing how the individual microbes survive with and without the host will uncover fundamental knowledge regarding factors that may regulate the assembly of microbial communities in different environmental conditions.

Here, we tested the variation in environmental tolerances for bacterial isolates from *N. vectensis* collected from natural habitats and how association with other bacteria or the cnidarian host may influence their survival. First, we analyzed the survival and growth of individual bacterial isolates from the natural environment across relevant abiotic stressors characteristic of estuarine environments. Second, we determined if these bacterial species survive within the host at these extreme temperatures and salinities. Lastly, we determined how these environmental bacterial isolates influence the survival of the anemone host exposed to sustained heat stress. Some data presented in this publication were formerly reported in a dissertation by Quinton Krueger [40].

## 2. Materials and Methods

### 2.1. Animal and Sediment Collection

Adult *N. vectensis* and water were collected from six estuaries along the coast of North America: Georgetown, South Carolina; Great Sippewissett Marsh, Massachusetts; Odiorne, New Hampshire; Saco, Maine; Crescent Beach, Nova Scotia; and Chezzetcook, Nova Scotia. Animals were collected from a 1-mm mesh filter and transferred into 50-mL conical tubes for transport back to the University of North Carolina at Charlotte (Charlotte, North Carolina, United States). These animals and the water they were collected from were used for isolations of bacteria (see Section 2.2).

### 2.2. Microbial Isolations and Sequencing

Animals and water collections (isolate ID nomenclature: A## and W##, respectively) were serially diluted in sterile phosphate-buffered saline (PBS) and spread on either marine broth (MB) agar or heart infusion (HI) agar (Criterion, C5831). *N. vectensis* were individually separated into 1.5-mL tubes, mechanically dissociated with a pestle, and homogenized via vortex. The plates were incubated overnight, and distinct morphological colonies were selected for isolation. Bacterial isolates were taxonomically identified via 16S rRNA gene sequencing. DNA was extracted from 196 isolated colonies with Promega Wizard Genomic DNA Purification kit, and the 16S rRNA gene was amplified using Q5 Mastermix (New England Biolabs) with the 27F forward and 1492R reverse primers. The amplicons were directly sequenced with Sanger sequencing (Eurofins). Resulting sequences were matched with the highest similarity to 16S rRNA gene sequences in the SILVA database [41].

Isolates from this study were compared with sequenced bacteria from a previous study of *N. vectensis* collected from the field and acclimated to the laboratory [7]. Sequences were aligned using clustal-omega v1.2.4 [42]. Due to the large number of unique sequences, both lab and field-associated amplicon sequence variants from this former study were truncated to the 100 most abundant sequences [7]. After alignment, a phylogenetic tree was constructed using maximum likelihood in IQTREE2 v.2.3.4 [43]. This tree was then imported into R v4.4.2 with the ggtree program v.3.14.0 and stylized [44].

### 2.3. Growth Curve of Isolates

Isolated bacteria from diverse taxonomic groups identified from the 16S rRNA gene data were selected for growth analysis under temperature and salinity conditions indicative of the range of conditions from the estuaries. Of 196 original isolates, 62 were selected for growth assays (Supporting Information 3: Table S1). These isolates were selected to be distributed among locations and taxonomic classifications. Selected isolates were from each location and seven genera for a wide representation of the culturable bacteria community. The conditions tested were three salinities and three temperatures in a full factorial design. For salinity, we used ⅓ Estuarine Broth (EB), EB, MB. The calculated salinities of ⅓ EB, EB, and MB are 5, 15, and 30 parts per thousand (ppt), respectively. For temperature, we used three high but environmentally relevant temperatures for these estuary environments: 30°C, 35°C, and 40°C [37]. The BioTek LogPhase 600 (Agilent Technologies) was used to conduct growth assays. We assayed six replicates per bacterial isolate for each tested salinity and temperature with optical density (OD) measured every 20 min for 48 h. Cultures were acclimated to the specific growth conditions overnight before inoculations in new media for the growth curves. These cultures were run in a shaking incubator at the specific temperature and appropriate media in 96-well plates with 149-*μ*L media and 1-*μ*L inoculum. After the overnight acclimation, 1 *μ*L was transferred to 149 *μ*L of fresh media, and the OD at 600 nm was measured through the time course in the LogPhase 600. The growth rate of each isolate (OD h^−1^) was calculated through the average of the six replicates followed by a log transformation and linear regression of the resulting data. Statistical comparisons of growth rates were performed with the nonparametric Kruskal–Wallis test. Significant differences were determined with a threshold of *p* < 0.05.

### 2.4. Analysis of Microbial Growth Assay (AMiGA) Analysis

The AMiGA pipeline was utilized for area under the curve (AUC) analysis of the growth curves generated from the BioTek LogPhase 600 [45]. First, individual runs were concatenated to a single file for batch analysis. Growth curves were exported from the LogPhase 600 and imported into the AMiGA pipeline. In AMiGA, the AUC was calculated for all growth curves. This, in combination of the growth rates, was used for PCA analysis in R. The PCA was visualized with the package ggplot2. To determine statistical significance, the function adonis from the vegan package was used for a PERMANOVA. Significant differences were determined with a threshold of *p* < 0.05.

### 2.5. *Nematostella vectensis* Cultivation

Adult anemones were isolated from the general laboratory population in the Reitzel Lab (University of North Carolina at Charlotte). To create axenic individuals, the polyps were exposed to the antibiotic cocktail (50 *μ*g mL−^1^ ampicillin [Acros Organics Cat. No. 61177], chloramphenicol [VWR, Cat. No. 0230], kanamycin [Fisher BioReagents, Cat. No. BP906], and neomycin [Alfa Aesar, Cat. No. J61499] each) for 24 h. Two hours before experimental exposures, anemones were washed with and transferred to sterile 15 ppt ASW.

### 2.6. Host Inoculation With Isolates

*Bacillus velezensis*, *Pseudoalteromonas spiralis*, and *Vibrio diabolicus* were inoculated into sterile seawater containing axenic adult *N. vectensis* to determine how these bacteria grow over time when associated with the animal. Five days before the thermal and bacterial, if applicable, exposures, *N. vectensis* adults were isolated to the respective salinities and then exposed to the antibiotic cocktail, if applicable, for 24 h. Two hours before bacterial exposures, all organisms were washed with 30 mL of their respective sterile ASW (5, 15, or 30 ppt). Bacteria were cultured as described in Section 2.2. Each day after the initial inoculation, five anemones were sampled using previous methods [46] to determine the number of colony-forming units (CFUs). Statistical comparisons of CFU counts were performed with the nonparametric Kruskal–Wallis test. Significant differences were determined with a threshold of *p* < 0.05.

### 2.7. Isolate Stability in Hosts Experiencing Abiotic Stressors

Adult *N. vectensis* were treated with the antibiotic cocktail for 24 h to reduce the laboratory microbial community. *N. vectensis* adults were then washed with 30 mL of sterile ASW to remove residual antibiotics. Two hours after the wash, anemones were exposed to 10^8^ CFUs of bacteria in sterile 15 ppt ASW. After 10 min of exposure, animals were washed with 30-mL sterile ASW animals and then isolated into to 96-well plates (VWR, 10861-562). Over 56 h, *N. vectensis* was exposed to three 8-h thermal spikes at 30°C or 40°C using a thermocycler (Bio-Rad T100). The details of the temperature profiles are provided in Supporting Information 3: Table S2. At the end of each cycle, five anemones were used to quantify CFUs, as previously described [46]. To identify isolate stability in ASW, individual bacteria were grown to 10^8^ CFUs mL^−1^ and diluted to 10^4^ CFUs mL^−1^ into either 5 or 30 ppt sterile ASW. CFU counts were performed after each thermal spike. Statistical comparisons of CFU counts were performed with the nonparametric Kruskal–Wallis test. Significant differences were determined with a threshold of *p* < 0.05.

### 2.8. Microbial Persistence in the Host During Long-Term Thermal Stress

To determine if the addition of specific bacteria influenced thermal tolerance of the anemone host, axenic *N. vectensis* juveniles seeded with the three bacteria (described in Section 2.6) were exposed to thermal gradients. Adult *N. vectensis* were exposed to bacteria and incubated for 10 min. Following bacterial exposure, juvenile anemones (*n* = 3, 12 individuals per temperature per replicate, nine temperatures) were isolated into individual 96-well plates. Plates were sealed (Breathe Easy covers) and then incubated in a thermocycler (Bio-Rad T100) at the following temperatures (36.2°C, 36.7°C, 37°C, 37.3°C, 37.8°C, 38.6°C, 39.5°C, 40.2°C, and 40.8°C) for 6 h. After thermal exposure, the animals remained at 20°C for 2 days and were assessed for mortality. Mortality was determined by either (1) deterioration of tissues or (2) no reflex to stimuli through observation under a stereoscope (Leica M80). To normalize the mean lethal temperature 50 (LT_50_) calculations, first, the means were transformed using the rank sum method. Following this transformation, the Shapiro–Wilk normality test results in a *p* value of 0.02, compared to the nontransformed data at *p* = 4.2e − 4. Therefore, we performed a nonparametric Kruskal–Wallis test, with Bonferroni adjustment for multiple comparisons.

## 3. Results

### 3.1. Phylogenetic Analysis of Bacterial Isolates

**Figure 1:**
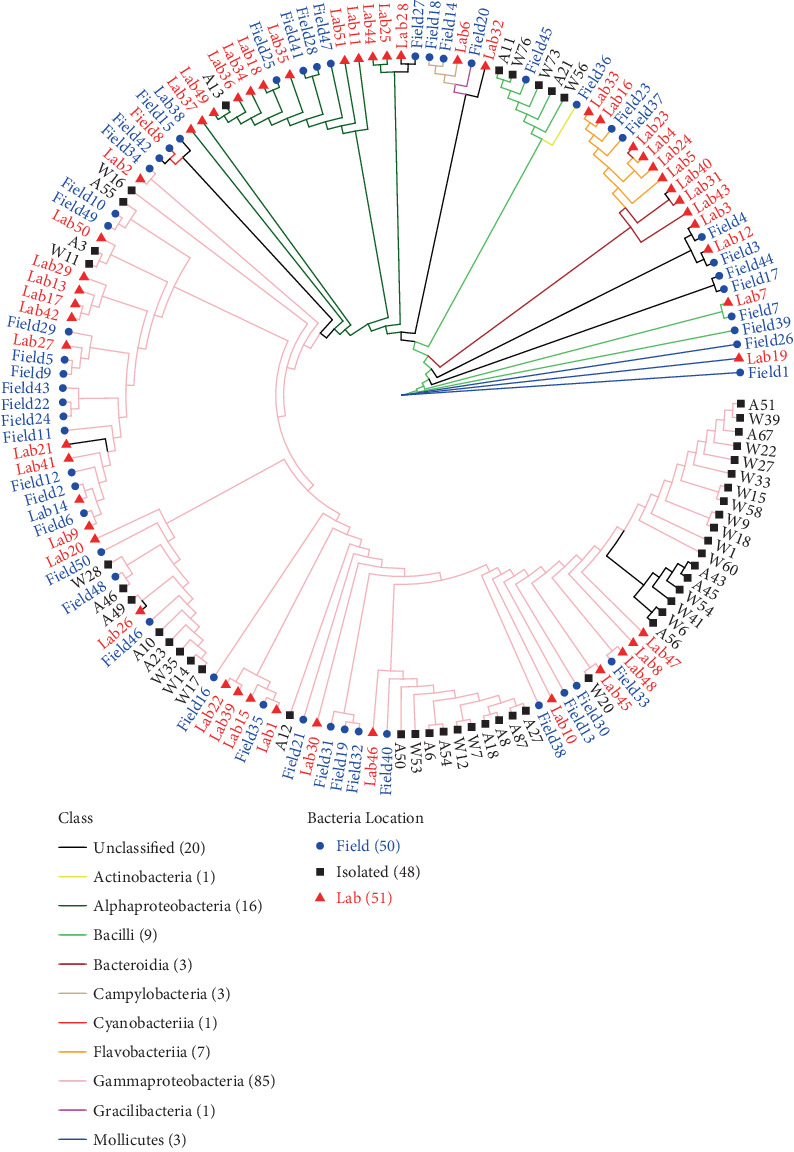
Phylogenetic analysis of the 62 bacterial isolates collected from *N. vectensis* and the surrounding water. 16S rDNA sequences generated from this study have black text. The field and lab-associated sequences from Baldassare et al. [7] have blue and red text, respectively. The number associated with the field and lab labels denotes the rank position of abundance for that specific ASV in each respective dataset. The color of the lines in the cladogram represents the taxonomic class of organism.

We sequenced a majority of the 16S rRNA gene from 48 bacterial isolates (14 failed to produce sufficient sequence) from field samples of *N. vectensis* and its estuarine environment. After alignment of the sequences to previously published 16S rRNA gene data by Baldassare et al. [7], the best fit model calculated by IQTREE2 was the TVMe+G4 model according to the Bayesian information criterion. Following ML analysis with bootstrap calculations, the bacterial isolates from the *N. vectensis* field animals grouped with most clades found in Baldassare et al. [7] (Figure 1). The cultured bacteria grouped within the *Alteromonas*, *Bacillus*, *Grimontia*, *Photobacterium*, *Pseudoalteromonas*, *Shewanella*, and *Vibrio* genera, where collection sites spanned five different estuaries along the east coast of the United States (Supporting Information 3: Table S3). Of the remaining isolates, the isolated bacteria grouped with previously described bacteria including *Exiguobacterium*, *Jonesia*, *Nitratireductor*, *Psychrobacter*, *Ruegeria*, and *Tritonibacter* (Supporting Information 1: Figure S1). Many of the isolates grouped within the Gammaproteobacteria order, for example, *Vibrio* and *Pseudoalteromonas*, whether they were isolated from both the host organism and the surrounding estuarine water. Using different media types, we were able to isolate rarer bacteria. For example, soil-derived plates (SOIL) isolated *Rossellomorea*, and higher salinity media (40 ppt) cultured *Exiguobacterium* and *Nitratireductor* (Supporting Information 1: Figure S1).

### 3.2. Growth Rates

**Figure 2:**
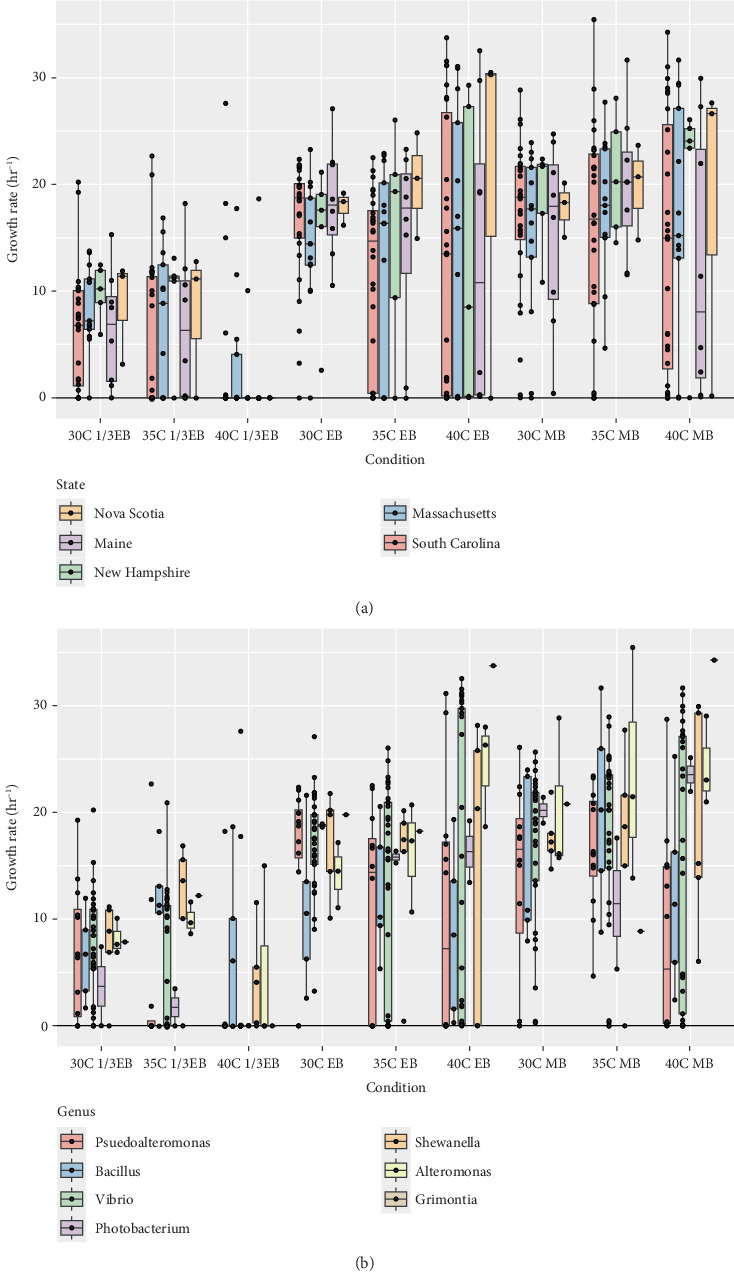
Mean growth rates of isolated bacteria under variable conditions. The *y*-axis represents the growth rate (h^−1^), and the *x*-axis is the conditions grouped by temperature and salinity. The whisker bar plots represent the distribution of each group, and the dots are the mean growth rate for each individual isolate. (a) Mean growth rate grouped by the location of the estuary from which the bacteria were isolated by state. (b) Mean growth rate grouped by the genus of the bacteria.

Sixty-two isolates from geographically distinct estuaries along the east coast of the United States were assessed for growth at variable saline and temperature conditions. The calculated growth rate (OD h^−1^) ranged from 0 h^−1^ (no detectable growth) to a maximum of 36.13 h^−1^. The highest temperature and lowest salinity (40°C and 5 ppt) were the most restrictive for the growth of the bacterial isolates. Isolates from the genera *Photobacterium* (0/3), *Vibrio* (0/15), and *Grimontia* (0/1) phyla were unable to grow under these conditions. Growth was also limited for most of the isolates from each location: South Carolina (3/27), Massachusetts (4/12), New Hampshire (1/5), Maine (1/7), and Nova Scotia (0/2) grew at 40°C and 5 ppt. For the other salinity and temperature conditions, bacteria generally grew during the 2-day incubation (Figure 2). When comparing growth rates for isolated bacteria from the five estuaries, we measured large variation between isolates depending on the salinity and temperature combination (Figure 2a). While few bacteria grew at the combination of high temperature and low salinity (see above), bacteria from the two higher latitude locations (New Hampshire and Nova Scotia) showed generally high growth rates at all temperatures in MB and 30°C and 35°C in EB. Bacteria isolated from South Carolina and Massachusetts showed larger variation in growth rates, including a number of isolates with low growth across these abiotic conditions. When we compared growth rates based on the taxonomic group of the bacterial isolates, we measured similarly large variation within and between groups (Figure 2b). For most groups, isolates had high growth rates or failed to grow under most abiotic conditions. This variation was particularly clear for the 40°C in both EB and MB. The highest growth rate for any bacteria isolated was for a *Vibrio* sp. (isolate ID W53) isolated from Maine when cultured under the 30°C 15 ppt condition (Table 1). This was followed by *Alteromonas* spp. (W15) from South Carolina at 35°C 30 ppt and then *Grimontia* spp. (W7) isolated from South Carolina under the same conditions.

Condition (*p* = 2.2e − 16), location (*p* = 0.03), and genus (*p* = 4.2e − 4) were all significant factors that impacted the mean growth rates of the bacteria. Twenty of the condition comparisons were statistically significant, while none of the location comparisons were statistically significant in the post hoc analysis. When comparing growth rate means by groups of genus, we found statistically significant differences between *Pseudoalteromonas–Alteromonas* (*p* = 2.2e − 3) and *Shewanella–Pseudoalteromonas* (*p* = 0.034), where *Pseudoalteromonas* isolates typically had lower growth rates. Furthermore, the interaction effect of location: genus (*p* = 3.4e − 5) also had a significant impact on the growth rates. When comparing the location: genus interaction terms, six comparisons were statistically significant, South Carolina: *Vibrio–*Nova Scotia: *Vibrio* (*p* = 0.025) and South Carolina: *Vibrio–*New Hampshire: *Vibrio* (*p* = 0.033), where the mean growth rates for *Vibrio* isolates from South Carolina were significantly lower than the other two locations.

### 3.3. Bacterial Plate Counts

#### 3.3.1. Artificial Seawater, Temperature, and Bacterial Growth

**Figure 3:**
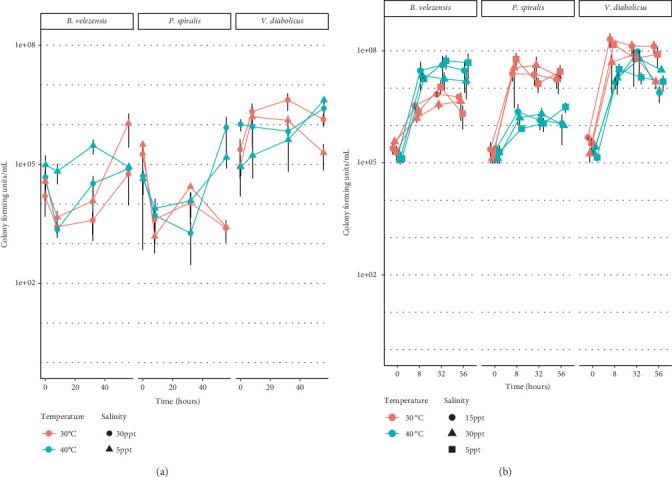
Plate counts of the three individual bacterial isolates. (a) Bacteria were exposed to extended static thermal exposure across saline gradients in sterile ASW over 3 days. (b) Bacteria were exposed to extended static thermal exposure across saline gradients in nutrient-rich media over 3 days. The *y*-axis represents the number of colony-forming units (CFUs) for each bacterial species, with time represented on the *x*-axis. The color of the points and lines represents temperature, while salinity is indicated by shape. Error lines represent standard deviation.

Individual isolates were inoculated at 10^5^ CFUs mL^−1^ into artificial seawater and cultured for 56 h (Figure 3a). Salinity was not significantly related to the concentration of bacteria in artificial seawater over the time course. Temperature was a significant factor for the concentration of bacteria in artificial seawater (*p* = 0.0373). Interestingly, *V. diabolicus* concentrations of bacteria were significantly higher when compared with the two other bacteria, while there was not a significant difference in the concentrations of *P. spiralis* and *B. velezensis* (*p* = 0.1011). All timepoint comparisons were not significantly different from each other except for the 8 and 56 h (*p* = 0.0001). For all the comparisons between conditions for an individual species, no conditions were statistically significant from each other. At 30°C and 5 ppt, *V. diabolicus* had a significantly higher concentration than both *B. velezensis* and *P. spiralis*. *V. diabolicus* also had the highest growth at 30°C and 30 ppt ASW.

#### 3.3.2. Nutrients, Temperature, and Bacterial Growth

Individual bacteria (*B. velezensis*, *P. spiralis*, and *V. diabolicus*) were inoculated at 10^5^ CFUs mL^−1^ (Figure 3b) with media and cultured for 56 h to compare with data from the LogPhase. *V. diabolicus* increased growth for 8 h that exceeded the concentrations of both *B. velezensis* and *P. spiralis* under all tested conditions. Furthermore, *V. diabolicus* maintained a higher concentration through 56 h when compared to *B. velezensis* and *P. spiralis* at all salinities and temperatures. Neither temperature nor salinity was a significant factor for growth in nutrient-rich media (*p* = 0.1075 and *p* = 0.8737, respectively). Bacterial species was a significant factor for the different bacterial plate counts when cultured in nutrient media (*p* < 2e − 16). *Pseudoalteromonas spiralis* and *B. velezensis* were not statistically different from one another (*p* = 0.991). *Vibrio diabolicus* CFUs were significantly higher than both *P. spiralis* and *B. velezensis*.

### 3.4. Bacteria In Vivo *Nematostella vectensis*

#### 3.4.1. No Antibiotic Treatment: Plate Counts

**Figure 4:**
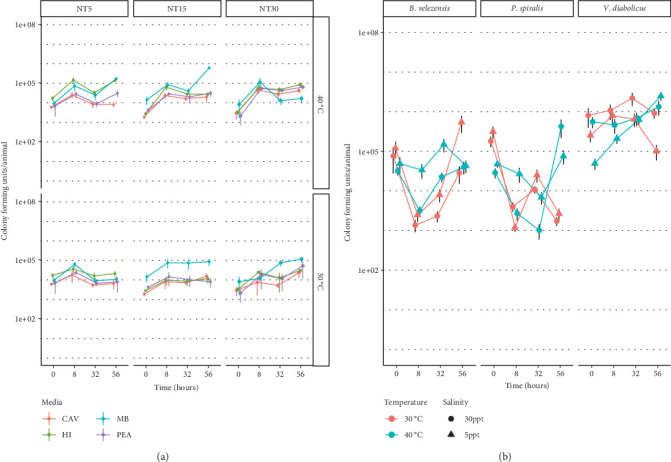
Plate counts of the three individual bacterial isolates exposed to extended static thermal exposure across saline gradients in axenic *Nematostella vectensis* over 3 days. The *y*-axis represents the number of colony-forming units (CFUs) for bacteria, with time represented on the *x*-axis. The color of the points and lines represents temperature, while salinity is indicated by shape. Error lines represent standard deviation. (a) NT conditions, CFUs were measured on marine broth (MB), heart infusion (HI), ChromAgar Vibrio (CAV), and phenylethyl alcohol (PEA) agar media. (b) Thermal spikes increased to 30°C or 40°C, and organisms were inoculated with either *B. velezensis*, *P. spiralis*, or *V. alginolyticus*.

Individual anemones survived the 40°C fluctuating condition but were visibly stressed, which was observed by limited response to tactile stimulation. Animals that were antibiotically treated did not yield colony growth (data not shown). For no antibiotic treatment (NT) anemones, the number of cultivated bacteria was statistically significant at 30°C and 40°C with most conditions (*p* = 6.85e − 18, Figure 4a). The concentration of bacteria in *N. vectensis* at all times was significantly different, except for the 8- and 56-h timepoints. Alternatively, the salinity of the water did not significantly impact the concentrations of bacteria within the host. While salinity did not influence the concentration of bacteria, the type of media used to culture bacteria was a significant factor (*p* = 4.47e − 29). The post hoc Tukey's honestly significant difference indicates that all media type comparisons were statistically significant, where the nonselective medias, MB and HI, cultured higher concentrations of bacteria, while phenylethyl alcohol (PEA) and ChromAgar Vibrio (CAV) had lower CFU counts. Lastly, the interaction of temperature and time was statistically significant (*p* = 8.14e − 07). While the number of bacteria at the beginning of the time course was not significantly different between temperatures, all remaining timepoints at 8, 32, and 56 h were significant between 30°C and 40°C.

#### 3.4.2. Bacteria Add-Back: Plate Counts

Salinity alone did not have a significant impact on the number of added back bacteria that remained associated with the host organism (*p* = 0.85, Figure 4b). Moreover, the temperature was not a significant factor on the overall concentrations of bacteria in the host (*p* = 0.057). However, the concentration of the bacteria significantly differed over time (*p* = 6.47e − 14). All timepoints were significantly different from each other, except the last two time points, 32 and 56 h (*p* = 0.947). Additionally, the species of bacteria differentiated the cell concentrations in the host organism (*p* = 2.56e − 48). *Vibrio diabolicus* was significantly higher in concentration than the two other bacteria (*p* = 0), while the concentrations of *P. spiralis* and *B. velezensis* were not significantly different from each other (*p* = 0.988). The time and temperature interaction was statistically significant (*p* = 1.46e − 06). Interestingly, the concentration of bacteria in the 40°C condition was not significantly different over time when compared to the initial concentrations. Alternatively, the concentrations between *t* = 0 at 30°C were all higher and significantly different from 8, 32, and 56 h (*p* = 0, *p* = 3.70e − 05, and *p* = 2.64e − 08, respectively).

*Bacillus velezensis* changed in concentration relative to the initial concentration over 8 and 32 h (*p* = 5.55e − 08 and *p* = 0.046, respectively) but returned to the initial concentration after 56 h (*p* = 0.366). *Pseudoalteromonas spiralis* had significantly different concentrations of bacteria over time when compared to the beginning of the time course. The concentration of *V. diabolicus* did not change throughout the time course when compared to the original starting concentration. The starting concentration of *V. diabolicus* was significantly higher than *B. velezensis* (*p* = 0.031) but remained similar to *P. spiralis* (*p* = 0.957). Throughout the time course, *B. velezensis* and *P. spiralis* maintained similar concentrations at all remaining time points. Moreover, the concentration of *V. diabolicus* was significantly higher than both other bacteria over the time course. The Tukey's post hoc analysis indicates that salinity was a significant factor for the concentration of *V. diabolicus*, where the higher 30 ppt salinity tended to have higher *V. diabolicus* CFUs in the host (*p* = 0.037). For both *B. velezensis* and *P. spiralis*, salinity did not influence the concentration of the bacteria in the host (*p* = 0.702 and *p* = 0.430, respectively).

#### 3.4.3. Anemone Resilience to Temperature With Added Bacteria

**Figure 5:**
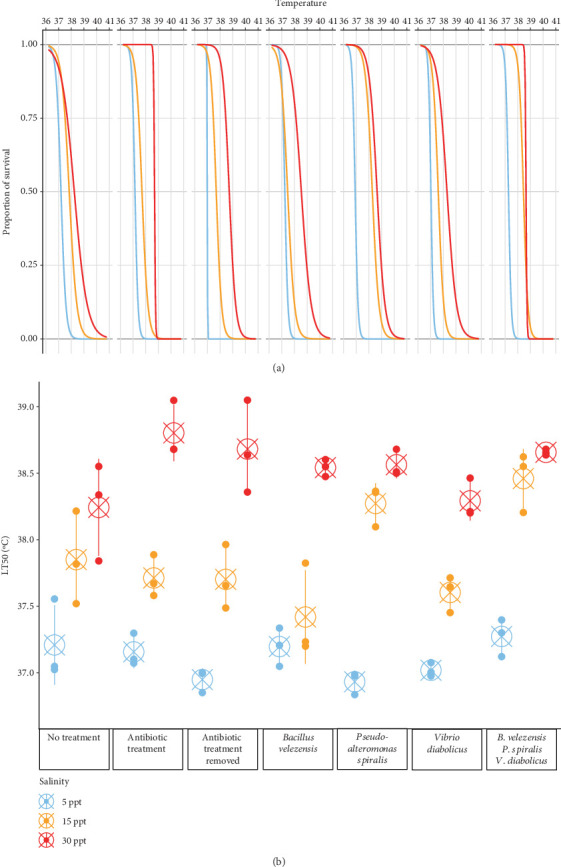
Lethal temperature 50s (LT_50_) of *Nematostella vectensis* under different treatment conditions. *Nematostella vectensis* LT50 measurements (*n* = 3, 12 individuals per temperature per replicate). Dots represent the mean LT50 of each replicate, while circles with crosses represent the mean across replicates, and error bars represent standard deviation.

*Nematostella vectensis* (*n* = 3, 12 individuals per temperature per replicate, nine temperatures) was exposed to 6 h of sustained elevated temperatures to determine if the microbiome impacts thermotolerance (Figure 5). Salinity was a significant factor in determining the LT_50_ of *N. vectensis*. Generally, the LT_50_ was higher with the increase in salinity. Five parts per thousand ASW had the lowest LT_50_ values with respect to all bacterial conditions compared to 15 and 30 ppt ASW. Additionally, the highest salinity ASW conditions had the highest mean LT_50_ values. Several variations in the microbiome resulted in significant differences in the LT_50_ of the host organism. Both the no treatment and antibiotic-treated anemones had significantly different LT_50_ values between the 5 and 30 ppt conditions (*p* = 1.00e − 4 and *p* = 0, respectively), where anemones in the higher salinity survived at higher temperatures. The antibiotic removed condition had significant differences in LT_50_ between all three salinities. When *B. velezensis* was added back to axenic anemones, there was a significant increase in the LT_50_ of the host, between 5 and 30 ppt and between 15 and 30 ppt (*p* = 0 and *p* = 2.00e − 4, respectively). For *P. spiralis*, there was a significant increase in survival in the host compared to 5 ppt and the other two salinities. *Vibrio diabolicus* inoculation had no significant difference between 5 and 15 ppt (*p* = 0.524), while there was a significant increase in host LT_50_ between 5 and 30 ppt (*p* = 0). Additionally, the anemones with three bacteria seeded in combination had significantly increased LT_50_ when comparing the 5 ppt saline condition to the higher salinities. *Bacillus velezensis* and *P. spiralis* inoculations at 15 ppt were also significantly different (*p* = 0.0471), where inoculated *P. spiralis* resulted in a higher host LT_50_. The hosts that were inoculated with three bacteria at 15 ppt had higher survival than many of the other microbiome conditions, where the antibiotic treatment removed had no increase when compared to the no treatment controls (*p* = 0.0888), while the *B. velezensis* and *V. diabolicus* artificial microbial communities significantly increased the mean LT_50_ of *N. vectensis* (*p* = 1.00e − 4 and *p* = 0.0199, respectively).

## 4. Discussion

In these studies, we focused on identifying and characterizing diverse culturable bacteria from the sea anemone *Nematostella vectensis* and its estuarine habitats to better understand how they may contribute to the microbial community of this species. We found that the animal-associated bacteria isolated from the environment represented a subset of the bacteria identified with 16S rRNA gene amplicon sequencing in animals that originated from the laboratory or the natural environment [7]. Thus, while the microbiome of field and laboratory-acclimated anemones certainly differs [7], the taxonomic diversity for these communities can be cultured with a combination of media types. *Vibrio*, other Gammaproteobacteria, and Firmicutes are amenable to laboratory culture [47, 48], which may explain the higher presence of isolates from *N. vectensis* and estuaries. Interestingly, we isolated several *Bacillus* species, which may serve as additional candidate probiotic organisms [49, 50], though we did not observe clear probiotic effects for the one *Bacillus* species we tested in the host anemone. We found that different substrates can isolate distinct and rare bacteria, as we have demonstrated with nontraditional media types, such as media derived solely from the organic compounds in soil and high salinity media. Using specific media types will continue to be useful to isolate and independently characterize the microorganisms that constitute the microbiome.

After testing an array of environmentally relevant conditions for phylogenetically diverse bacterial isolates, both salinity and temperature were major influences that impacted the growth rate and survivability of the culturable bacteria. The combination of these abiotic factors was particularly influential in growth when at low salinity and high temperature. Comparatively, in another study characterizing isolated bacteria from *N. vectensis*, while not the same genus, showed similar thresholds for the temperature and salinity of their selected isolates [33]. This range of temperatures as a singular stressor began limiting growth at 37°C, while salinity could range from 0 to 50 ppt, though optimal was 20–30 ppt [33]. The bacteria characterized in this study were isolated from a broad range of estuaries along the eastern coast of North America, ranging from South Carolina to Nova Scotia. Interestingly, the location that the bacteria were isolated from was a significant factor in the growth rate of phylogenetically similar bacteria. This highlights the importance of determining the individual growth and physiological properties of bacteria and their location of isolation, as opposed to their phylogenetic classification. The temperatures of eastern North American estuaries can fluctuate upwards of 20°C daily, and the minimum and maximum can be from −5°C to 42°C, respectively [37, 51]. While these temperatures are permissive for growth and sustainability of diverse microbiomes [8], the combination of high temperature and low salinity can be limiting for bacterial growth. *Pseudoalteromonas spiralis* (W20) showed the highest growth among the tested conditions, and it is possible that this isolate could outcompete pathogenic bacteria, for example, *Vibrio coralliilyticus* in similar conditions. *Pseudoalteromonas* sp. have shown antagonistic properties against pathogens isolated from marine species [52, 53], including sponges [54] and corals [55]. Given these environmental fluctuations, optimizing probiotic and antagonistic bacteria based on their phenotypic growth over extreme conditions may improve host health.

Salinity is a significant abiotic factor that could impact coastal invertebrates and their microbiomes. For instance, higher salinity is positively correlated with host thermal tolerance, including *Exaptaisia* [56] and corals [57, 58]. Additionally, acclimation to different salinities, which results in the shifting of microbial communities, can confer thermal tolerance [4, 59], which is supported by the Becking Baas theory [12]. This shift in thermal tolerance could also be explained by the individual physiology of the host [56], the bacteria that constitute the community [60], or both [59]. Here, we found that lower salinities resulted in low or no growth for the bacteria that were isolated from the natural environment. This is supported by increased physiological stress of low salinity in cnidarians, including bleaching caused by low salinity in corals [61, 62]. Previous research has shown that modest changes to the salinity of *N. vectensis* significantly impact the microbial community [35], along with other cnidarians [4]. Estuary salinities can vary due to location but also vary with low salinities due to an influx of freshwater from rainfall. *N. vectensis* is reported to reside in estuaries that vary in salinity from 2 to 52 ppt [63]. The data shown here support the strong effect salinity has as a stressor for individual bacteria, especially in conjunction with other abiotic stressors, including temperature.

In this study, we observed large fluctuations in concentrations of bacteria under changing temperatures. *Nematostella vectensis* that were exposed to the smaller thermal fluctuations resulted in minimal shifts in culturable CFUs over the time course. In the higher temperature exposures, the bacterial CFUs present inside the host fluctuated daily. This finding agrees with previous research, where the relative number of bacteria within *N. vectensis* can fluctuate over short time scales [34]. While the lower temperature conditions resulted in low fluctuations of culturable CFUs throughout the time course, this number remained consistent at both 30°C and 40°C, which is a potential indicator of microbiome stability. Alternatively, when single isolates are introduced into the host, elevated temperatures create instability for the individual bacteria, thus resulting in large fluctuations in concentration. This supports the idea of engineering complete microbiomes, as opposed to the addition of individual isolates, when influencing the composition of the associated microbial community and ultimately host stability [64]. Few studies have quantified the culturable number of CFUs in cnidarians [18, 46, 65]. For example, the transmission methods of solution-mediated and prey-mediated bacteria impact the number of bacteria that associate with the host [46]. Determination of isolate stability is vital for effective implementation of methodologies for inoculating and measuring host-associated bacteria and their interactions with the host.

*Vibrio diabolicus*, *B. velezensis*, and *P. spiralis* were all successfully reinoculated into the host, and their numbers fluctuated over short, environmentally relevant thermal spikes. Additionally, we found that when these bacteria are introduced into the axenic host, they readily associated with and maintained association with the animal. Over a longer time course, if the native microbiome is present in the host, specific isolates that are transferred into the organism are lost [64]. We found that these three bacteria can be differentially retained in axenic organisms over the time course. The dominant bacteria in our trials was *V. diabolicus*, a related species to documented pathogens [11, 66–68]. At elevated temperatures, we found an increased concentration of *V. diabolicus* at both tested salinities in the host. This result indicates the importance of microbiome maintenance, as pathogens may associate with the host prior to probiotic application, thus reducing or eliminating colonizing probiotics. The individual physiological tolerance of potential probiotics must be characterized prior to field use to effectively determine if the isolated bacteria can survive the physiological stress of the environmental system the host inhabits.

Previous research with *N. vectensis* has shown that the microbiome can serve a protective role in higher temperature environments [7, 8]. Here, we found that salinity, bacterial species, and the combination of these factors can have an influence on the thermal tolerance (LT_50_) of this species as well. *N. vectensis* has been collected from estuaries with large differences in salinity (2–52 ppt), with the highest numbers of individuals in salinities from 16 to 32 ppt [63] The effect of salinity could be attributed to physiological variation of the anemone in different saline conditions (as shown with the axenic, antibiotic removed condition). Alternatively, inoculated bacteria increased the LT_50_ when compared to both axenic and no treatment controls at 15 ppt. Moreover, we found that the combination of three coinoculated bacteria increased the LT_50_ to a greater degree than the individual bacteria, which indicates the currently adopted method of adding monoculture probiotics may not be as effective as adding a combination of bacteria, as they all may contribute to the survival of the animal under thermal and saline stress. *P. spiralis* was incapable of growth at 5 ppt and 40°C and had reduced growth at 35°C. These high temperature conditions that reduce and ultimately eliminate growth for *P. spiralis* do not improve the survivability of *N. vectensis* to long-term thermal stress, which is comparable to the axenic conditions at 5 ppt. Furthermore, *Bacillus velezensis* had reduced growth at 15 ppt, but its growth characteristics remained stable in culture at the same conditions over 48 h. While gram-positive *Bacillus* has been proposed as an effective probiotic for heat-stressed organisms [69–71], we found that at 15 ppt the best performing thermal tolerant bacteria was *P. spiralis*. This indicates that the direct bacterial–host interactions may also play a role in the survivability of the host under multiple stressors, which requires additional investigation.

The inoculation of beneficial bacteria has been shown to produce favorable outcomes in diseased marine invertebrates [29, 72, 73]. While this is the ideal outcome, the individual physiological capabilities of these inoculums should be determined prior to their use in host organisms. For example, when bacteria are inoculated in *N. vectensis*, this specific inoculum can be lost within 7 days [64]. Furthermore, multiple inoculating bacterial species may prove to be the ideal approach, as species such as those found in *Bacillus* genera can promote the enrichment of other potentially probiotic bacteria [28]. Additionally, we found that inoculation of nonpathogenic *Vibrio* can marginally reduce the overall thermal tolerance of the host. This is illustrated in many marine invertebrate systems, including corals [74–76], mussels [31, 77], sea urchins [78], and oysters [24, 79], where *Vibrio* spp. can increase host susceptibility to abiotic factors (e.g. saline and thermal stress), which is correlated to the relative abundance of *Vibrio* [80]. Candidate bacteria for use *in vivo* should be phenotypically characterized prior to their use in marine invertebrates to determine the potential outcomes of stressed organisms and whether the bacteria that is reintroduced into the marine organisms could have a direct impact on the survival of the host.

## 5. Conclusion

Microbiomes are diverse communities of bacteria that are associated with animals and other organisms. Here, we experimentally show the wide range of growth conditions that individual bacteria have in these associated communities and how this may drive the changes in the animals' microbiome in variable environments. Using inoculation studies with three focal bacteria, we show that the host has a relatively small impact on extending the environmental conditions that the bacteria can survive in. However, the addition of particular bacteria can significantly impact the survival of the host. Overall, the growth and survival characteristics of individual bacteria in addition to the composition of the bacteria naturally associated with a host need to be considered for the interpretation of changes in the microbiome and how it may impact physiologically stressed organisms.

## Figures and Tables

**Table 1:** Top three growth rates of bacterial isolates derived from Nematostella vectensis, or surrounding estuarine water, per condition. The isolate ID, genus, and location of the estuary from which the bacteria were derived. Growth rates are reported as h^−1^.

**Condition**	**Isolate ID**	**Genus**	**Location**	**h ** ^**−1**^
40°C MB	W7	*Grimontia*	South Carolina	34.27
	W66	*Shewanella*	Massachusetts	33.43
	W58	*Vibrio*	Maine	32.01

35°C MB	W15	*Alteromonas*	South Carolina	35.46
	W76	*Bacillus*	Maine	31.66
	W1	*Vibrio*	South Carolina	28.94

30°C MB	W55	*Vibrio*	Maine	30.73
	W12	*Alteromonas*	South Carolina	28.85
	W54	*Pseudoalteromonas*	Maine	28.51

40°C EB	W7	*Grimontia*	South Carolina	33.76
	A45	*Vibrio*	Maine	32.54
	W18	*Vibrio*	South Carolina	31.55

35°C EB	W22	*Pseudoalteromonas*	South Carolina	26.55
	A67	*Vibrio*	New Hampshire	26.03
	A50	*Vibrio*	Nova Scotia	24.84

30°C EB	W53	*Vibrio*	Maine	36.13
	W6	*Vibrio*	South Carolina	29.79
	W60	*Vibrio*	Maine	27.10

40°C 1/3EB	W76	*Bacillus*	Maine	18.65
	W20	*Pseudoalteromonas*	South Carolina	18.22
	A25	*Vibrio*	Massachusetts	17.74

35°C 1/3EB	W20	*Pseudoalteromonas*	South Carolina	22.66
	A6	*Vibrio*	South Carolina	20.90
	W76	*Bacillus*	Maine	18.21

30°C 1/3EB	A6	*Vibrio*	South Carolina	20.22
	W20	*Pseudoalteromonas*	South Carolina	19.27
	W41	*Shewanella*	Massachusetts	14.71

## Data Availability

The data that supports the findings of this study are available in the supplementary material of this article.

## Supporting Information

Supporting Information 1 Figure S1. Isolated bacteria from *Nematostella vectensis* from field and lab populations.

Supporting Information 2 Figure S2. Principal component analysis of the 62 isolates' growth capabilities across thermal and saline conditions. Ellipses represent 95% confidence intervals.

Supporting Information 3 Table S1. Distribution of microbial isolates selected for growth assays. Table S2. Temperature profiles for thermocycler experiments at 30°C and 40°C. Table S3. Distribution of isolates collected from estuaries along the eastern coast of the United States. Isolates were clustered by genus and by northern sites (ME, NH, NS, and MA) and southern site SC. ME = Maine, NH = New Hampshire, NS = Nova Scotia, MA = Massachusetts, SC = South Carolina.

Supporting Information 4 Figure_1.fasta. This file contains the sequences generated from this work, in addition to the truncated lab and field ASV sequences from Baldassare et al. [7]. These sequences were used to generate Figure 1.

Supporting Information 5 S_Figure_1.fasta. This file contains the sequences generated from this work, in addition to other bacteria that were isolated from *N. vectensis*.

Supporting Information 6 Summary_stats.xlsx. File containing summary statistics for experiments.
