# Supplementary figures and images for: Influence of Temperature and Saline Conditions on Bacteria Naturally Associated With the Cnidarian Host Nematostella vectensis
Source: Int J Microbiol. 2025 Nov 21;2025:4107949. doi: 10.1155/ijm/4107949 (PMC12662692; doi:10.1155/ijm/4107949)

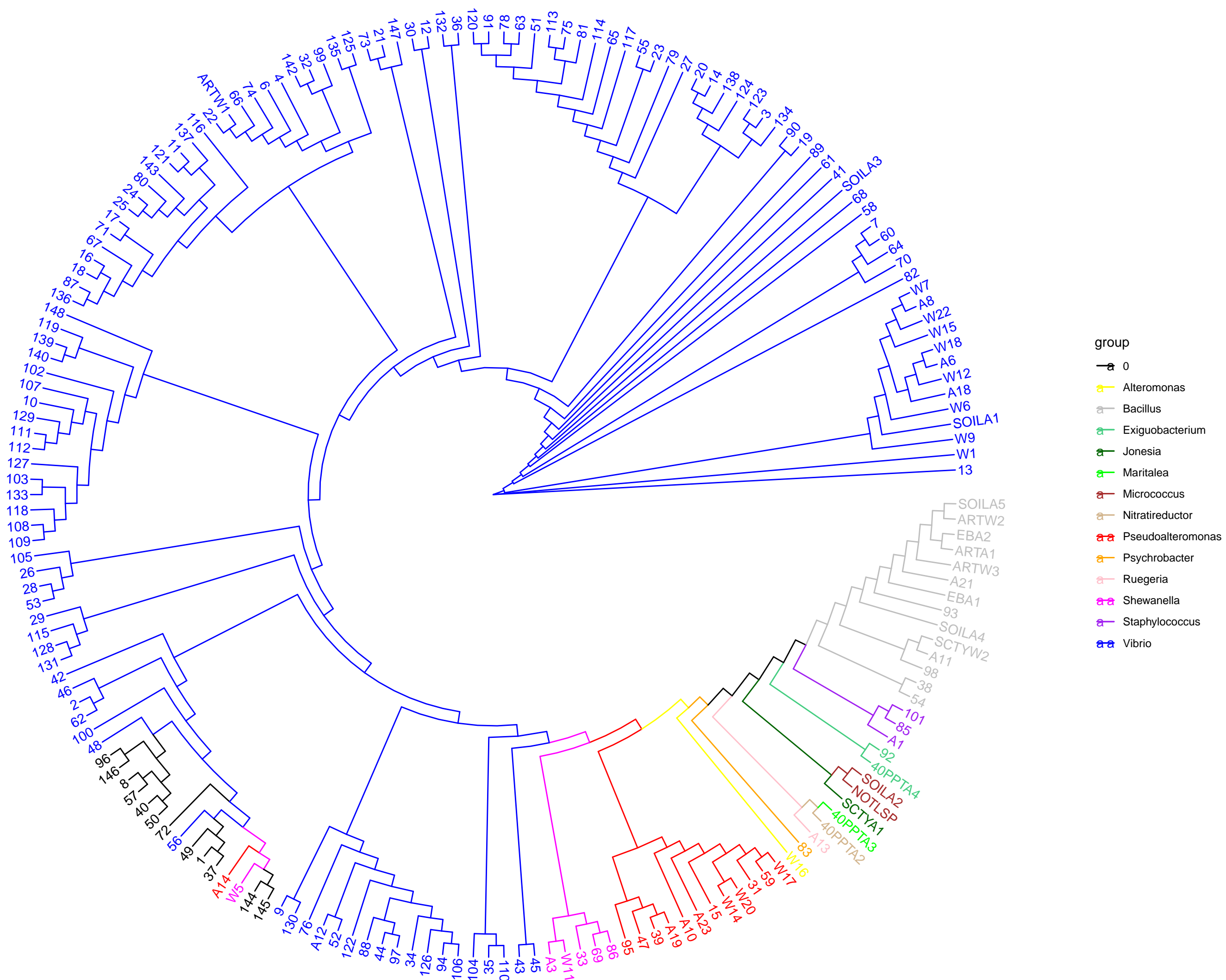

Supplement: Supporting Information 1 — Figure S1. Isolated bacteria from Nematostella vectensis from field and lab populations. [file 4107949.f1.pdf]

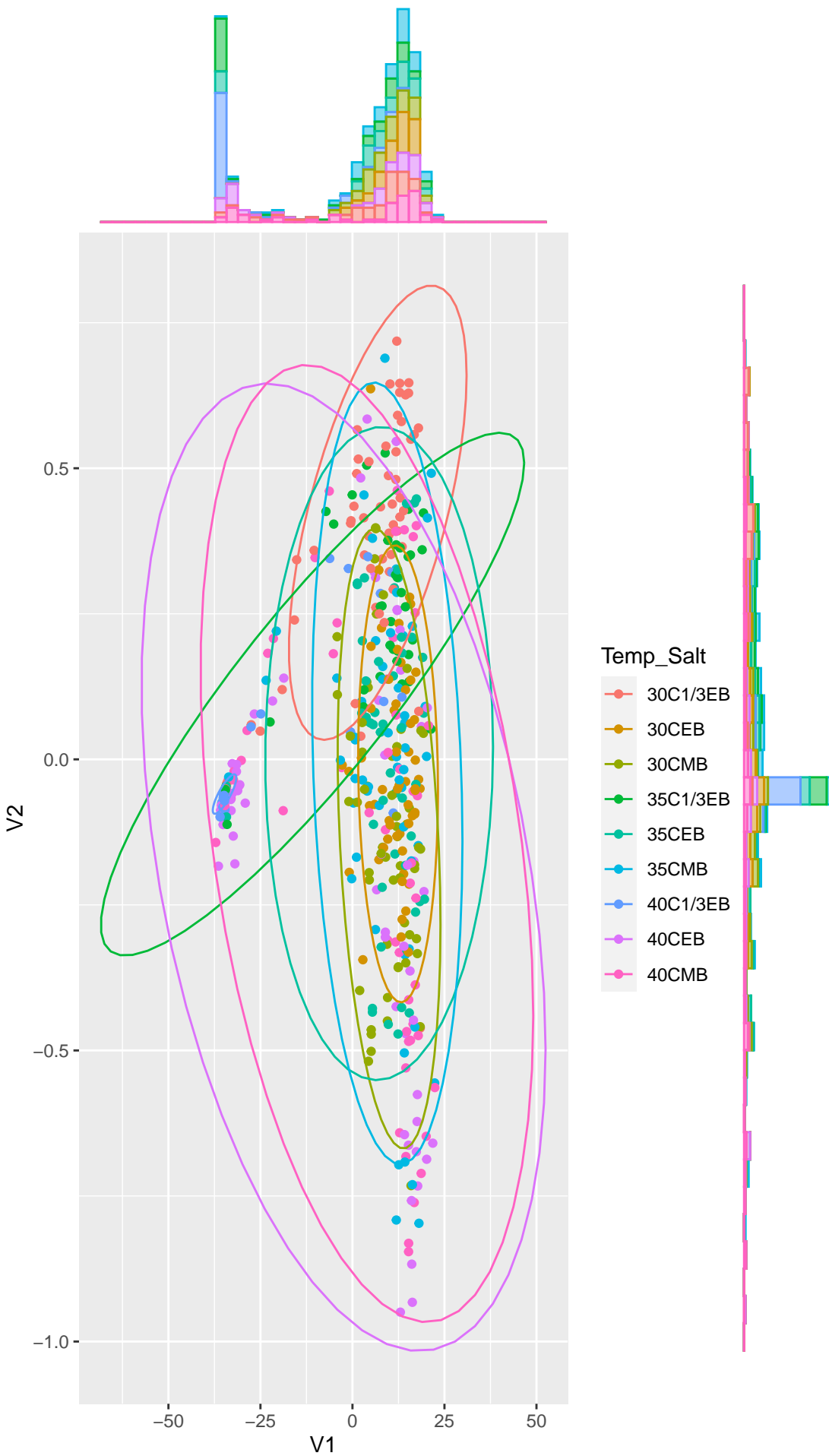

Supplement: Supporting Information 2 — Figure S2. Principal component analysis of the 62 isolates' growth capabilities across thermal and saline conditions. Ellipses represent 95% confidence intervals. [file 4107949.f2.pdf]
