# Supplementary material for: Influence of Temperature and Saline Conditions on Bacteria Naturally Associated With the Cnidarian Host Nematostella vectensis
Source: Int J Microbiol. 2025 Nov 21;2025:4107949. doi: 10.1155/ijm/4107949 (PMC12662692; doi:10.1155/ijm/4107949)
Supplement: Supporting Information 3 — Table S1. Distribution of microbial isolates selected for growth assays. Table S2. Temperature profiles for thermocycler experiments at 30°C and 40°C. Table S3. Distribution of isolates collected from estuaries along the eastern coast of the United States. Isolates were clustered by genus and by northern sites (ME, NH, NS, and MA) and southern site SC. ME = Maine, NH = New Hampshire, NS = Nova Scotia, MA = Massachusetts, SC = South Carolina. [file 4107949.f3.docx]

| Isolate composition | Nova Scotia | Maine | New Hampshire | Massachusetts | South Carolina |
| --- | --- | --- | --- | --- | --- |
| *Alteromonas* |  |  |  |  | 3 |
| *Bacillus* |  | 2 | 1 |  | 3 |
| *Grimontia* |  |  |  |  | 1 |
| *Photobacterium* |  | 1 |  |  | 1 |
| *Pseudoalteromonas* | 1 | 1 |  | 3 | 6 |
| *Shewanella* |  |  |  | 3 | 2 |
| *Vibrio* | 3 | 9 | 2 | 7 | 13 |

Supplemental Table 1: Distribution of microbial isolates selected for growth assays.

| 40°C | 25°C | 30°C | 35°C | 40°C | 35°C | 30°C | 25°C | 20°C |
| --- | --- | --- | --- | --- | --- | --- | --- | --- |
| 30°C | 22.5°C | 25°C | 27.5°C | 30°C | 27.5°C | 25°C | 22.5°C | 20°C |
| Step Duration (h) | 1 | 1 | 1 | 1 | 1 | 1 | 1 | 17 |
| Total Duration (h) | 1 | 2 | 3 | 4 | 5 | 6 | 7 | 24 |

Supplemental Table 2. Temperature profiles for thermocycler experiments at 30°C and 40°C.

|  | ME NH NS MA | | SC | |
| --- | --- | --- | --- | --- |
| Genus | Found | Selected | Found | Selected |
| *Agarivorans* | 0 | 0 | 2 | 1 |
| *Shewanella* | 3 | 3 | 2 | 2 |
| *Vibrio* | 119 | 14 | 24 | 13 |
| *Alteromonas* | 0 | 0 | 4 | 3 |
| *Pseudoalteromonas* | 7 | 7 | 10 | 7 |
| *Bacillus* | 4 | 4 | 4 | 3 |
| *Grimontia* | 0 | 0 | 1 | 1 |
| *Photobacterium* | 0 | 0 | 1 | 1 |
| *Exiguobacterium* | 1 | 0 | 0 | 0 |
| *Psychrobacter* | 1 | 0 | 0 | 0 |
| Total | 148 | 31 | 48 | 31 |

Supplemental Table 3. Distribution of isolates collected from estuaries along the eastern coast of the United States. Isolates were clustered by genus, and by northern sites (ME, NH, NS, and MA) and southern site SC. ME = Maine, NH = New Hampshire, NS = Nova Scotia, MA = Massachusetts, SC = South Carolina.
